# Zn$_{1-x}$Ni$_x$Te semiconductor nanocrystals in transparent glass for optoelectronic device applications

**DOI:** 10.1038/s41598-023-34591-0

**Published:** 2023-05-10

**Authors:** Radha Mada, Hamid Darabian, Seshadri Meruva, Maria José V. Bell, Alessandra S. Silva, Noélio O. Dantas, Virgílio C. Anjos

**Affiliations:** 1 Grupo de Engenharia e Espectroscopia de Materiais, Departamento de Física, Universidade Federal de Juiz de Fora, Juiz de Fora, MG 36036-900 Brazil; 2 Laboratório de Novos Materiais Nanoestruturados e Funcionais, Instituto de Física, Universidade Federal de Alagoas, Maceió, AL 57072-900 Brazil

**Keywords:** Lasers, LEDs and light sources, Optical spectroscopy

## Abstract

Doping glass with semiconductors, particularly with nanostructured semiconductors, has attracted attention due to the large optical absorption cross-sections of the latter. Based on this property, Ni$^{2+}$ (5 wt%) doped phosphate glass and Zn$_{1-x}$Ni$_x$Te (x = 0.5, 1.0, 5.0 and 10.0 wt% of Ni$^{2+}$) nanocrystals (NCs) doped phosphate glasses (GCs) were prepared by fusion method and subsequent heat treatment. Influence of Ni$^{2+}$ on structural, thermo-optical and third-order nonlinear optical properties have been analysed through various spectroscopic characterizations. The XRD pattern of the glass (G) exhibits the amorphous nature of the host material while GCs exhibit not only amorphous halo but also the presence of quantum dots (QDs) or nanocrystals (NCs) phases. TEM analysis of the studied GCs samples confirm the presence of quantum dots (QDs) and bulk NCs with an average diameter of approximately 4.2 ${\pm }$ 0.3 nm and 13.4 ${\pm }$ 0.2 nm, respectively. Several phosphate groups were observed and reported from Raman and FTIR-ATR spectra. The absorption band positions confirmed that Ni$^{2+}$ ions resemble to the octahedral symmetry. The intensity of absorption band around 1352 nm ($^3$T${_1}$(F) $\rightarrow$ $^3$A${_2}$(F)) increased with the increase of Ni$^{2+}$ in GCs which is an indicative of the $^{[6]}$Ni$^{2+}$ coordination. The emission properties such as emission cross-sections (${\sigma }_{emi}$) full width at half maxima (FWHM) for the $^1$T${_2}$(D) $\rightarrow$ $^3$T${_2}$(F) (visible) and $^3$A${_2}$(F) $\rightarrow$ $^3$T${_1}$(F) (near-infrared) emission transitions were reported. Among the glass-containing semiconductor nanocrystals (GCs), the emission cross-sections in GC4 sample (x = 10% of Ni$^{2+}$) are the largest for both the visible (11.88 $\times$ 10$^{-18}$ cm$^2$) and infrared (0.98 $\times$ 10$^{-20}$ cm$^2$) transitions. Thermal diffusivity (D), thermal conductivity (K) and temperature dependent optical path length change (ds/dT) were obtained through time-resolved thermal lens (TL) and thermal relaxation (TR) methods. The D and K parameters do not change significantly with increase of Ni$^{2+}$ ions (0.5–5%) in GCs. Nonlinear-refractive index and nonlinear absorption of the studied samples were also obtained using femtosecond Z-scan technique. The increase of nonlinear absorption coefficient ($\beta$) is observed from GC2 (2.53 ${\times }$ 10$^{-10}$ cm/W) to GC4 (7.98 ${\times }$ 10$^{-10}$ cm/W). The GC4, sample with 10 wt% of Ni$^{2+}$, showed the lowest ds/dT (1.22 $\times$ 10$^{-6}$ K$^{-1}$) with good lasing (FOM and emission cross-sections) and nonlinear absorption properties suggesting that it can be a good candidate for visible-red emission light conversion in LED technology.

## Introduction

Semiconductor materials with combination of III-V type (GaAs, InAs and GaN), II-VI type (ZnO, ZnTe, CdTe, ZnS), and IV (Si, Sn and Ge) have been the focus of continued attention since they provide applications in the field of optoelectronics^1^, solar photovoltaic cells^2^, photodetectors^3^ and, diluted magnetic semiconductors (DMS)^4^ when transition metals are incorporated in some binary semiconductors. Concerning DMS for spintronic applications, the addition of magnetic transition metals and/or rare earth ions to the II-VI semiconductors are interesting due to their greater solubility limit and lower concentration of defects^5^. The incorporation of a magnetic transition metal ion into the semiconductors leads to a strong s-d interaction between the carriers and the local magnetic ions that may modify the spin splitting and the spin polarization properties. ZnTe is an important II-VI semiconductor with a wide and direct band gap of 2.26 eV at room temperature with applications in optoelectronic and thermoelectric devices^6,7^. Its recent integration with DMS materials in various forms (bulk, thin film or nanostructured) has been object of investigation concerning their structural, electrical and magnetic behavior^6,8,9^.

The addition of nickel (Ni) to a glass may exhibit stable Ni$^{2+}$ ions which favors large crystal field stabilization energy at an octahedral environment^10^. These ions have strong visible (Vis) and near-infrared (NIR) absorption bands originated from the electronic ground state of Ni$^{2+}$ in octahedral symmetry, $^3$A$_2$(F) to the excited states, $^3$T$_2$(3F), $^3$T$_1$(3F) and $^3$T$_1$(3P)^11^. Numerous researches have been devoted to investigating the optical properties of Ni-doped phosphate glasses^12–14^. In particular, P$_2$O$_5$ acts as a glass networking former and the structure is based on the corner sharing PO$_{4}$ tetrahedron which form chains, rings or isolated PO$_{4}$ groups. Phosphate glasses are technologically important due to some particular properties such as high thermal expansion coefficient, low melting and softening temperatures, high ultraviolet transmission, and low phonon energy over conventional silicate and borate glasses^14^. Currently, magneto-optical properties in phosphate glasses via magnetic transition ions and semiconductor nanocrystals doping have been performed, although, the reports have been limited to structural and magnetic properties^15–18^. Moreover, so far the thermo-optical properties of ZnTe based DMS in phosphate glass are not reported. These properties are required to systematic studies with suitable magnetic dopant and concentration for applications in magnetic and/or optical properties (optical saturation).

In this work, the effect of Ni$^{2+}$ ions in structural and thermo-optical properties of transparent P$_2$O$_5$-ZnO-Al$_2$O$_3$-BaO-PbO glass (G) and the same glass containing semiconductor nanocrystals (GC) were studied through DTA, XRD, TEM, IR absorption and Raman spectroscopy, visible and near-infrared absorption, thermal-lens and z-scan techniques. Our results gave hints about optimal composition eligible to visible-red emission light conversion in LED technology.

## Results and discussion

### X-ray diffraction and structural analysis

**Figure 1:**
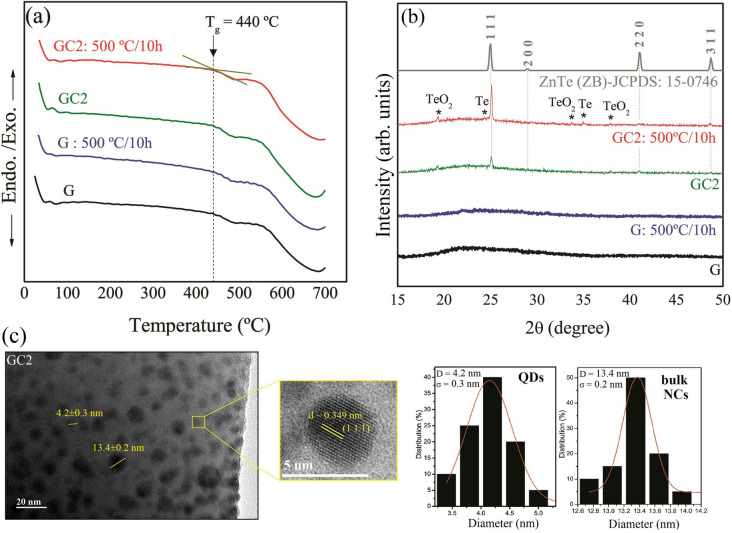
(**a**) DTA thermogram (**b**) XRD diffractogram of G (5 wt% Ni$^{2+}$ ions in glass) and GC2 glass sample (1 wt% Te & x = 1 wt% Ni$^{2+}$ ions doped) without heat treatment and heat treated at 500 $^{\circ }$C for 10 h; (**c**) TEM image of GC2 (heat treated at 500 $^{\circ }$C for 10 h) glass sample along with amplification of selected region of the image and histograms for QDs and bulk NCs (right side).

Figure 1a shows the DTA thermograms of samples G and GC2 without heat treatment and with heat treatement at 500 $^{\circ }$C for 10 h. The value of the glass transition temperature is around Tg = 440 $^{\circ }$C, and this value did not undergo significant changes with the heat treatment or with the presence of NCs in the host glass. This suggests that the structural changes are small or insignificant in the glass lattices, since there are no well-defined crystallization peaks when the samples were heat treated or doped with NCs. Given these results, it can be concluded that the glasses are thermally stable at room temperature and at temperatures around T$_g$. They also have high mechanical strength and can be applied as active medium for lasers. In addition, given the value of T$_g$ = 440 $^{\circ }$C, it was possible to confirm that the temperature of 500 $^{\circ }$C (a little above T$_g$) is completely adequate for the heat treatment of the glass samples. This temperature is capable of promoting the diffusion of precursor ions Zn$^{2+}$, Te$^{2-}$ and Ni$^{2+}$, favoring the formation and growth of NCs and QDs. Figure 1b shows the XRD pattern of the same samples presented in Fig. 1a^19^. It can be seen that two broad humps at around 15 < 2$\theta$ < 30 and 35 < 2$\theta$ < 50° indicate the amorphous nature of the host glass. The G sample clearly exhibits glassy characteristics by the presence of the diffuse hump and the absence of sharp peaks which would correspond to crystalline phase. The GC2 sample exhibits not only the amorphous halo, but also sharp diffraction peaks that can be attributed to the growth of quantum dots (QDs) or nanocrystals (NCs). The peaks related to reflections from 25.00 (1 1 1), 28.89 (2 0 0), 41.12 (2 2 0), and 48.59 (3 1 1) evince zinc blende structure and match the standard card of ZnTe, JCPDS: 15-0746^20^. The diffractograms also show an increase in the intensity of the main peak (111). This behavior is related to the increase of NCs density in the host vitreous matrix, with the thermal treatment. Other peaks observed in the diffractograms are related to constructive interferences from 19.30 (1 0 1), 33.60 (1 0 2) and 38.00 (2 0 0) of TeO$_2$ in a tetragonal form^21^ and from 24.43 (1 0 0) and 35.10 (1 0 2) of Te in a trigonal form^22^.

According to the literature^15–18,23^, doping the ZnTe host by different concentrations of transition metals (Ni, Mn, Co, etc.) does not induce changes in lattice positions, a fact that was confirmed by the transmission electron microscopy (TEM). Figure 1c exhibits TEM images of GC2 glass sample. This sample was compared with the other glasses containing only ZnTe NCs (Ni-undoped) confirming that the crystallinity of these NCs were not altered by Ni content^15,16,23^. The images show two distinct groups of spherical Zn$_{1-x}$Ni$_x$Te NCs. One is attributed to quantum dots (QDs) and the other to bulk NCs, as also observed in previous work^23^. The average diameters of these NCs are approximately D ${\sim }$ 4.2 nm for QDs and D $\sim$ 13.4 nm for bulk NCs, as shown by the size histogram. The size dispersion ($\sigma$) for QDs and bulk NCs are, respectively, 0.3 and 0.2 nm. The amplified region of the TEM image shows that the distance between the adjacent lattice fringes is 0.346 nm. This value is largely consistent with the value found in the literature for (1 1 1) lattice parameter (d) (0.350 nm) and corresponds to ZnTe NCs with zinc-blend (ZB) structure (JCPDF No. 15-0746). The d spacing, average diameter and size dispersion of the NCs were determined using ImageJ software to find the best fit of the height distributions from a Log-Normal function. In addition, to calculate the diameter, around 30 particles were considered and at least five images were acquired for the same sample. Due to the lack of long range order in glasses, x-ray diffraction analysis is limited to structural features regarding local structure and vibrational unit arrangements of nearest neighbors. Other structural features are determined by the use of Raman and MIR measurements in the studied samples^19,24^.

**Figure 2:**
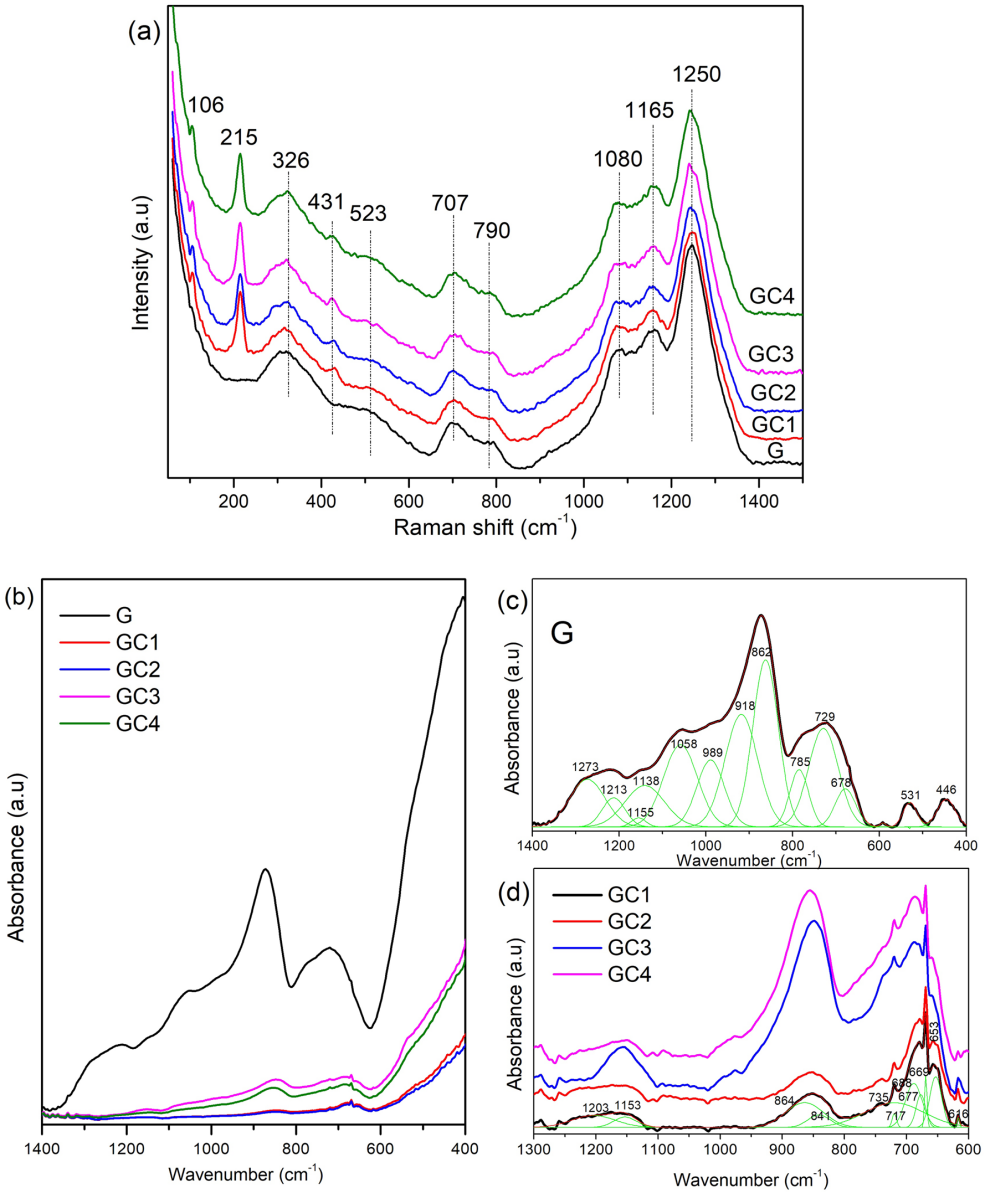
(**a**) Raman spectra, (**b**) IR absorbance spectra and (**c**,**d**) are the deconvolution spectra of the glass samples doped only with Ni$^{2+}$ ions and glass samples containing semiconductor nanocrystals.

Figure 2a shows Raman spectra for the glass samples doped only with Ni$^{2+}$ ions and glass samples containing semiconductor nanocrystals. The spectral profiles are similar for all samples. In the case of GC1 to GC4 samples, three new bands appear around 106, 215 and 431 cm$^{-1}$ being their natures attributed to the second order TO, first-order LO and second order LO phonon modes of ZnTe^25^ respectively. The band between 215 and 431 cm$^{-1}$ (326 cm$^{-1}$) is associated to the bending of PO$_4$ units^26^. The middle frequency region bands around 707 cm$^{-1}$ and 790 cm$^{-1}$ are asymmetric stretching of P-O-P bending in Q$^2$ group (from meta phosphate). In the higher frequency region bands around 1100 cm$^{-1}$ and 1165 cm$^{-1}$ are the symmetric stretching mode of P-O-P non-bridging oxygen bond in Q$^1$ and Q$^2$ groups^27^. The band around 1250 cm$^{-1}$ is due to the asymmetric stretching of P-O-P non-bridging oxygen bond in Q$^2$ groups^26,28,29^.

Figure 2b shows ATR-FTIR spectra for the Ni$^{2+}$ ions doped glass and glass-ceramic samples. As seen from the Fig. 2b, the IR absorption bands are broader (see Fig. 2c) than Raman bands and the identified bands reveal the following features. In the case of glass (G): (a) a broad band between 1175 cm$^{-1}$ and 1350 cm$^{-1}$; (b) doublets in the region between 1020 and 1175 cm$^{-1}$; (c) a strong intense band at 873 cm$^{-1}$; (d) A weaker band around 714 cm$^{-1}$. In the case of GCs: (a) a weak band around 1158 cm$^{-1}$; (b) an intense band 854 cm$^{-1}$; (c) a broad band between 625 and 795 cm$^{-1}$; and observed changes regarding intensity (decrease) and position (toward lower wavenumber side) of bands compared with G sample. The ZnTe vibrational modes at very low frequency (nearly 220 cm$^{-1}$) region are not shown because they are in the limit of measure range of the spectrophotometer.

In order to extract new information as the phosphate glasses contain many structural groups, a quantitative analysis was performed by the deconvolution of the IR absorption of Ni$^{2+}$ doped G and GC1 (see Fig. 2c,d). The deconvoluted spectra reveal a number of absorption bands that are attributed to various vibrational modes of phosphate structural groups. In order to perform deconvolution, we corrected the spectrum baseline using BRUKER Opus 6.0 software tool, and then chose a fixed peak position where the Gaussians match the measured spectra with different combinations of curves in different positions using Origin software through multi-peak fit. The fitted results showed a good Gaussian fit with R$^2 =$ 0.9988. From the Fig. 2c (G sample), the band at 446 cm$^{-1}$ is attributed to the bending vibration O-P-O units in PO$_4^{3-}$ groups (Q$^0$ units). The 531 cm$^{-1}$ band is related to the O-P-O bending vibrations in (P$_2$O$_7$)$^{4-}$ (Q$^1$ units)^29^. The bands 678, 728 and 785 cm$^{-1}$ are associated to the symmetric stretching vibration of P-O-P groups in Q$^1$ units^30^. The 862 cm$^{-1}$ and 918 cm$^{-1}$ bands are related to the asymmetric stretching vibration of P-O-P units in ${PO_4^{3-}}$ groups (Q$^0$ units)^29^. The 989 and 1058 cm$^{-1}$ bands are attributed to the symmetric and asymmetric stretching vibrations of ${PO{_4^{3-}}}$ groups (Q$^0$ units)^30^. The bands at 1138 and 1155 cm$^{-1}$ are due to symmetric stretching vibration of ${PO{_3^{2-}}}$ groups (Q$^1$ units) and ${PO^{2-}}$ groups (Q$^2$ units)^31^. The bands 1213 and 1273 cm$^{-1}$ are associated to symmetric and asymmetric stretching vibration of ${PO{_2^-}}$ groups (Q$^1$ units)^30^.

As seen from the Fig. 2d (GC samples), the appearance of a new band at 616 cm$^{-1}$ is due to a change of in-chain P-O-P groups by the effect of network modifier on phosphate glass structure^32^. The important band between 623 and 700 cm$^{-1}$ (P-O-P groups in Q$^1$ units) is resolved by the composition of four bands. It is interesting to note that the relative area of the bands decreased from 0.12 (688 cm$^{-1}$)–0.02 (669 cm$^{-1}$), which suggests that the 653, 669, 677 and 688 cm$^{-1}$ bands are associated to the bending vibration of P=O bonds, but not to the P-O-P groups^29^. The 717 and 735 cm$^{-1}$ bands are related to the metaphosphate units, Q1 (P-O-P)^30^. The bands 841 and 864 cm$^{-1}$ are due to the symmetric and asymmetric stretching vibration of P-O-P groups (Q$^0$ units)^31^. The 1153 and 1203 cm$^{-1}$ bands are related to the symmetric stretching vibration of ${PO{_2^-}}$ groups in Q$^2$ and Q$^1$ units^30^. Moreover, the intensity of 717 and 737 cm$^{-1}$ bands in GCs increase with the decrease/disappearance of the band around 1058 nm via the increase of Ni$^{2+}$ concentration. This result may be interpreted as a decrease of the pyrophosphate units with consequent increase of the metaphosphate units^31^.

### Optical absorption and emission spectroscopy

**Figure 3:**
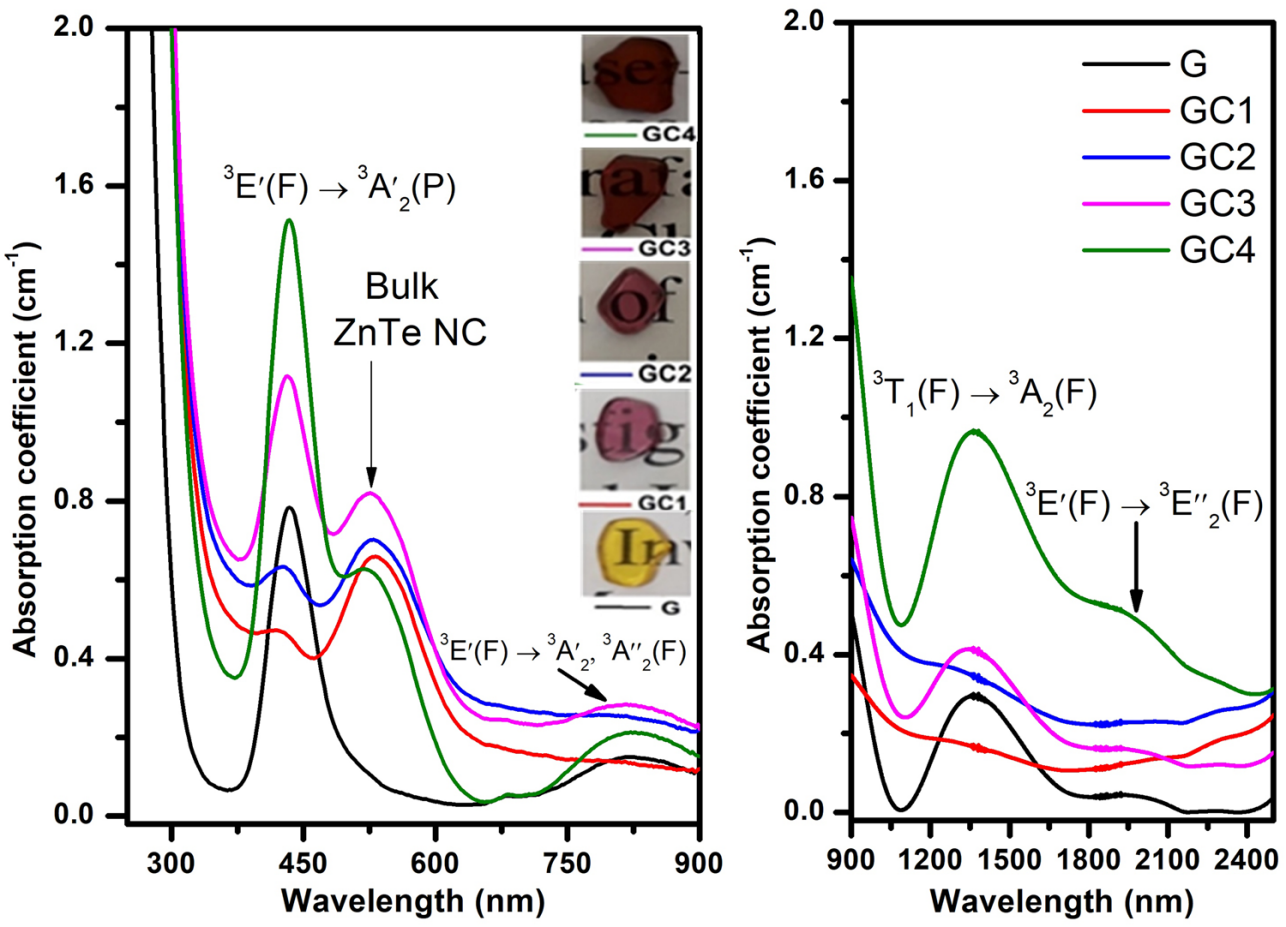
Optical absorption spectra of the glass samples doped only with Ni$^{2+}$ ions and glass samples containing semiconductor nanocrystals.

UV-Vis-NIR absorption spectra of the glass samples doped only with Ni$^{2+}$ ions and glass samples containing semiconductor nanocrystals are shown in Fig. 3^19^. The nature and position of the bands are associated to the fivefold coordinated Ni$^{2+}$ in trigonal pyramid ($^{[5]}$Ni$^{2+}$) and tetrahedral ($^{[4]}$Ni$^{2+}$) sites and bulk property of ZnTe-like NCs^16,33^. In the G sample absorption spectrum, the observed bands around 435, 826, and 1963 nm are the characteristic bands of fivefold coordinated Ni$^{2+}$ in trigonal pyramid ($^{[5]}$Ni$^{2+}$) sites, and the band around 1356 nm is associated to the tetrahedral ($^{[4]}$Ni$^{2+}$) sites. In the spectra of heat-treated samples, GC1 to GC4, the spectral pattern and four absorption band positions are similar to G sample (exception for GC1 and GC2 samples which present low-intensity absorption profiles in the near-infrared region). According to literature^33–35^, these absorption bands can be assigned to $^3$E′(F) $\rightarrow$ $^3$A′$_2$ (P) (435 nm), $^3$E′ (F) $\rightarrow$ $^3$A″$_1$, $^3$A″$_2$ (F) (826 nm), $^3$T$_1$ (F) $\rightarrow$ $^3$A$_2$ (F) (1356 nm) and $^3$E′ (F) $\rightarrow$ $^3$E″ (F) (1963 nm), respectively. In addition, a pronounced red shift of absorption edge observed in heat-treated samples indicates a liquid phase separation and the formation of ZnTe crystals. The formation of ZnTe crystals is clearly evident by the appearance of an absorption band around 533 nm, which is equal to the optical band gap of ZnTe (2.33 eV), in GC1 to GC4 samples. The increase in the intensity of $^{[5]}$Ni$^{2+}$ and $^{[4]}$Ni$^{2+}$ bands with the amount of nickel ions in GCs suggests spinodal decomposition (alternating regions of low and high concentration of magnetic impurities). However, we can not neglect the Ni$^{2+}$ ions partition between $^{[4]}$Ni$^{2+}$ and $^{[6]}$Ni$^{2+}$ sites due to a decrease of line-width of the fivefold coordinated Ni site around 435 nm ($^3$E′(F) $\rightarrow$ $^3$A′$_2$ (P)) and an absorption band ($\sim$ 400 nm ($^3$A$_2$(F) $\rightarrow$ $^3$T$_1$(F)) due to $^{[6]}$Ni$^{2+}$ species that are overlapped with the absorption edge^35^. Such coordination changes may lead to change in coloration of the samples (see Fig. 3).

**Table 1:** Absorption band energies $({\nu })$ and optical band gaps $(E_{opt})$ of G and GC glasses.

Sample	G	GC1	GC2	GC3	GC4
$^3T_1 (F) \rightarrow ^3A_2 (F)$ $(\text{cm}^{-1})$	7294	7289	7283	7283	7273
$^3E' (F) \rightarrow ^3A''_1, ^3A''_2 (F)$ $(\text{cm}^{-1})$	12,195	12,255	12,255	12,255	12,077
$^3E'(F) \rightarrow ^3A'_2 (P)$ $(\text{cm}^{-1})$	23,041	23,809	23,364	23,148	23,041
$E_{opt}$ (Direct) (eV)	4.55	4.13	4.15	4.16	4.14
$E_{opt}$ (Indirect) (eV)	3.81	3.75	3.77	3.72	3.72

The fundamental absorption edge in UV region of G is around 262 nm and is shifted to longer wavelength $({\sim }300 \; \text{nm})$ in GCs. The optical transitions such as direct and indirect occur at the fundamental absorption edges of crystalline and non-crystalline materials. According to Davis and Mott^36^ theory^37^, the optical band gap $(E_{opt})$ values are estimated by plotting (${\alpha }h{\nu }$)$^2$ and (${\alpha }h{\nu }$)$^{1/2}$ as a function of photon energy $(h{\nu })$ that are shown in Supplementary Material (see Fig. S1a,b). The ${E_{opt}}$ is obtained by linear extrapolation of absorption curve to the h${\nu }$ axis and are reported in Table 1. The ${E_{opt}}$ values are 4.55 eV (direct) and 3.81 eV (indirect) for G sample. In the case of GCs a negligible variation is observed in their ${E_{opt}}$ values due to nickel ions content. In addition, the decreasing of $E_{opt}$ from G to GCs indicates the increase of non-bridging oxygens (NBOs)^37^.

**Figure 4:**
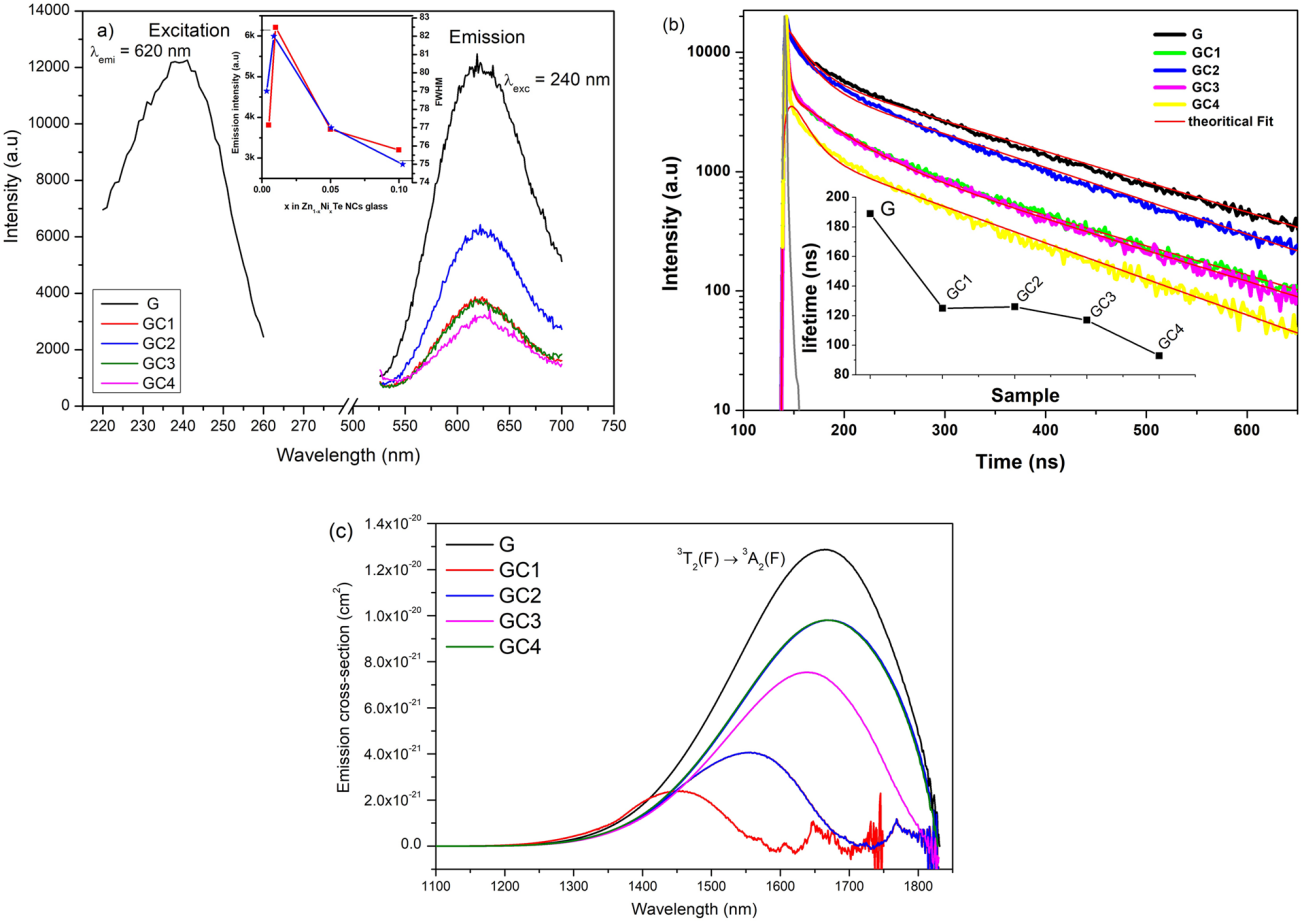
(**a**) Glass (G) and GCs vis-emission spectra together with the excitation spectrum. Inset: variation of emission intensity (red curve) and FWHM (blue curve). (**b**) Corresponding time-resolved decay curves. Inset: lifetime variation. (**c**) NIR emission cross-section spectra (from McCumbers theory) for glass samples doped only with Ni$^{2+}$ ions and glass samples containing semiconductor nanocrystals.

The excited levels of Ni$^{2+}$ ions vary with the change of host glass composition and their emission can be related to d-d optical transitions due to the existence of fivefold coordinated Ni$^{2+}$ in trigonal pyramid ($^{[5]}$Ni$^{2+}$) and tetrahedral ($^{[4]}$Ni$^{2+}$) coordination sites within the glass matrix. The emission spectra of 5% Ni doped and GCs are shown in Fig. 4a together with the excitation spectrum^19^. The excitation spectrum for the Ni doped glass was recorded by monitoring the emission at 620 nm. Under 240 nm excitation, the broad emission band between 525–700 nm wavelength is attributed to ${^1T_{2g}(D) \rightarrow ^3T_{2g}(F)}$ transition. The emission profiles are similar for the G and GCs but the intensity has decreased with the increase of the Ni$^{2+}$ concentration (x = 5 and 10 wt%) in GCs (see inset of Fig. 4a). In general, the excess of Ni$^{2+}$ ions which may escape from the host lattice form defects and trapping states that lead to increase of the non-radiative recombination and consequently to the decrease of luminescence intensity. The quenching of luminescence was also observed in Ni$^{2+}$ doped ZnS colloidal particles^38,39^. The full width at half maxima (FWHM) is found to be higher in GC2 (82 nm) and decreases with increasing of the nickel ions content in GCs (see inset of Fig. 4a).

Figure 4b shows emission decay curves for the visible emission transition, ${^1T_{2g}(D) \rightarrow ^3T_{2g}(F)}$ at 240 nm wavelength excitation^19^. The curves present good fit to bi-exponential decay (G and GC2) and tri-exponential decay (for GC1, GC3 and GC4) due to non-homogeneous distribution of the doping ions in the host glass. The use of two functions suggests that the carrier-relaxation mechanisms are dominated by the defect/trapping effects and bimolecular recombination mechanisms^40^. The average lifetimes are calculated using the following expressions:

1 $$\begin{aligned} \big \langle \tau \big \rangle = \frac{A_1{\tau _1^2}+A_2{\tau _2^2}}{A_1{\tau _1}+A_2{\tau _2}} \ \ and \ \ \big \langle \tau \big \rangle = \frac{A_1{\tau _1^2}+A_2{\tau _2^2}+A_3{\tau _3^2}}{A_1{\tau _1}+A_2{\tau _2}+A_3{\tau _3}} \end{aligned}$$

The inset of Fig. 4b shows the variation of lifetimes with Ni ions content in our glasses. The lifetime is saturated at 1.0% Ni$^{2+}$ ions (GC2) with subsequent decrease for higher doping concentrations. This could be the effect of environment change around the Ni$^{2+}$ ions or due to multipolar non-radiative interactions among Ni$^{2+}$ ions^41^. The lifetime for the GC3 (117 ns) has decreased compared with the G (188 ns). The variation in emission intensity, FWHM and decay lifetimes follow similar trends with increasing Ni$^{2+}$ in GCs which is a strong evidence of the change of environment surrounding Ni ions in the glasses.

**Table 2:** Emission properties of G and GC samples (* from McCumbers theory).

Sample	n	$^1T_2(D) \rightarrow ^3T_2(F)$					$^3A_2(F) \rightarrow ^3T_2(F)$	
		*FWHM* (nm)	${\Delta \lambda _{eff}}$ (nm)	${\tau _m}$ (ns)	${\sigma _{emi} \times 10^{-18}}$ $({\text{cm}^2})$	${FOM \times 10^{-25}}$ $({\text{cm}^2/\text{s}})$	${FWHM^*}$ (nm)	${\sigma _{emi} \times 10^{-20}}$ $({\text{cm}^2})$
G	1.515	90	90.38	189	5.01	9.48	256	1.29
GC1	1.526	79	78.94	125	8.57	10.70	169	0.24
GC2	1.531	82	83.54	127	7.94	10.04	204	0.41
GC3	1.543	77	76.39	117	9.24	10.81	252	0.75
GC4	1.535	75	75.45	93	11.88	11.06	278	0.87

The peak emission cross-section is calculated using the expression^37^,

2 $$\begin{aligned} \sigma _{emi}({\lambda }) = \frac{{\lambda }_p^4}{8{\pi }cn^2\tau _m\Delta \lambda _{eff}} \end{aligned}$$

where ${\lambda _P}$ is the peak wavelength, n is the refractive index, ${\Delta \lambda _{eff}}$ is the effective linewidth of the emission band and ${\tau _m}$ is the measured lifetime, respectively. Table 2 shows estimated ${\lambda _P}$, n, ${\tau _m}$, FWHM, ${\Delta \lambda _{eff}}$ and ${\sigma _{emi}}$ parameters. The effective linewidth $({\Delta \lambda _{eff}})$ is estimated dividing the area of the emission band by its maximum height. The emission cross-section is higher for GC4. The ${\sigma _{emi}}$ of 5 wt% Ni$^{2+}$ doped G ${(5.01\times 10^{-18} \; \text{cm}^2)}$ to GC ${(9.25\times 10^{-18} \; \text{cm}^2)}$ is significantly improved. The figure of merit (FOM) is an important parameter to characterize the laser materials and is calculated by the product of ${\sigma _{emi}}$ and ${\tau _m}$ since it is inversely proportional to the laser threshold and proportional to the gain amplification. The FOM is higher in GC4 ${(11.06\times 10^{-24} \; \text{cm}^2\,\text{s})}$ among the GCs.

Considering the emission: (i) the near infrared emission position of Ni$^{2+}$ ions are very sensitive to the crystal field strength, which might form structurally asymmetric shape profile; (ii) the Ni$^{2+}$ ions may occupy regular crystal lattices with strong crystal field strength when they are incorporated into the GCs, exhibiting broad emission ($^3$A$_2$(F) $\rightarrow$ $^3$T$_1$(F)) in near infrared region with small red shift compared with absorption. Therefore, the NIR emission of Ni$^{2+}$ may be reabsorbed by the neighboring Ni ions^42^. Figure 4c represents the emission cross-section spectra for near-infrared $^3$T$_2$(F) $\rightarrow$ $^3$A$_2$(F) transition which were calculated from the absorption spectra using McCumber’s reciprocity method^43^ and are listed in Table 2. The FWHM and emission cross-sections $(\sigma _{emi})$ and ${\tau _m}$ are found to increase with Ni$^{2+}$ ions in GCs and do not change significantly from G to GC3. The higher FWHM and $(\sigma _{emi})$ of $^3$T$_2$(F) $\rightarrow$ $^3$A$_2$(F) transition in GC4 sample (0.98 $\times$ 10$^{-20}$ cm$^2$) than that of reported matrices, i.e., Ni doped Li$_2$O-Ga$_2$O$_3$-SiO$_2$ glass-ceramics (0.63 $\times$ 10$^{-20}$ cm$^2$)^44^ and TBZLN tellurite glasses (Tm$^{3+}$: 0.52 $\times$ 10$^{-20}$ cm$^2$) and Ho$^{3+}$: 0.4 $\times$ 10$^{-20}$ cm$^2$)^43^, shows that this sample is suitable for broadband optical amplification in telecommunications operating at 1.1–1.8 μm region.

### Photo-thermal spectroscopy

**Figure 5:**
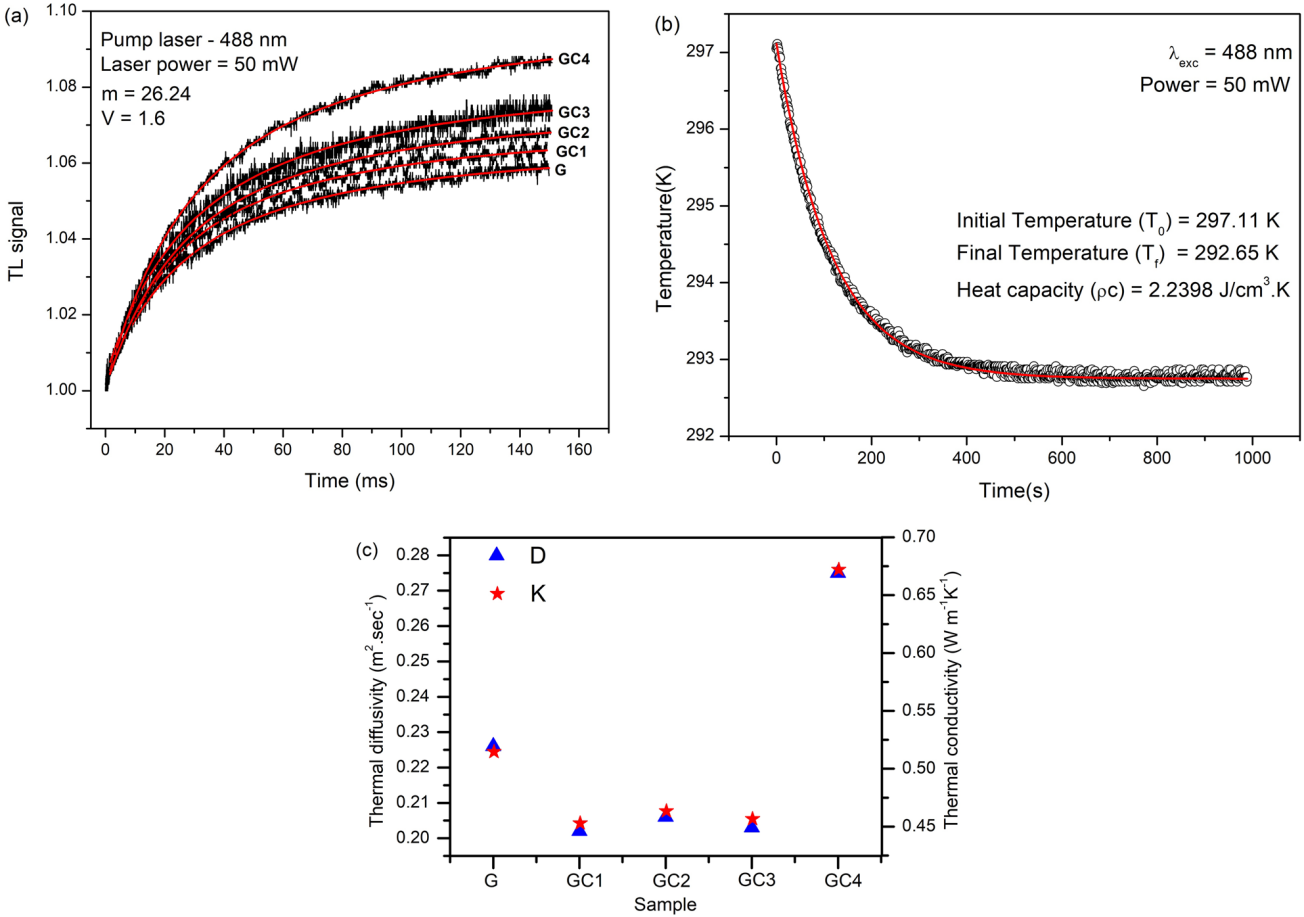
(**a**) Typical normalized TL signal for Ni doped glass. (**b**) Cooling curve of the specific heat capacity measurements for 5% Ni doped glass. (**c**) Shows variation of D and K with glass samples doped only with Ni$^{2+}$ ions and glass samples containing semiconductor nanocrystals.

It is known that the thermo-optical properties such as thermal diffusivity (D) and thermal conductivity (K) of glasses suffer significant variation based on thermal processing conditions, micro-structure and composition of the glass. Such properties are quite useful to determine the figure of merit (FOM) of optical glasses by knowing their potential to support thermal shock, thermal stress and thermal effects^45^. Therefore, D and K parameters are greatly affected by the accumulation of heat during the device operation and need to be reduced in the fabrication of a microelectronic device such as, radiation detectors, laser diodes, and transistors in electronic circuits. Among various methods, time-resolved thermal lens (TL) and thermal relaxation (TR) methods may be applied to a wide range of materials to determine thermal diffusivity (D), variation of optical path length with temperature (ds/dT) and thermal conductivity (K), respectively^45–47^. In this work, the D and ds/dT of Ni$^{2+}$ doped G and GCs were measured using TL experiment which is based on the two-beam mismatched mode. Details can be found elsewhere^45^. The excitation and probe beam radius at sample are ${\omega }_e$ = 40.78 μm and ${\omega }_p$ = 208.87 μm, respectively. Figure 5a shows TL transient signal of all samples with the excitation power of 50 mW. The observed shape of probe beam intensities indicates dS/dT>0, a divergent thermal lens. The solid lines represent theoretical fits using Eq. (1) given in Ref.^45^, resulting in ${\theta } = - (0.0655)$ and $t_c = (1.9652)$ ms. Using expression, $t_c={(\omega _e^2)/4D}$ , the thermal diffusivities (D) are calculated from the average of five measurements for each sample and are listed in Table 3.

**Table 3:** Thermo-optical properties of glass samples doped only with Ni$^{2+}$ ions and glass samples containing semiconductor nanocrystals.

Sample	$A_e$ ($(\text{cm}^{-1})$)	$t_c$ (ms)	$D {\times } 10^{-3}$ ($(\text{cm}^2/\text{s})$)	${\rho }c$ $(\text{J}/\text{cem}^3\; \text{K})$	$K {\times } 10^{-3}$ (W/cm K)	$dS/dT {\times }1 0^{-6}$ ($\text{K}^{-1}$)
G	1.1571	1.8521	2.2668	2.2701	5.1454	1.6746
GC1	2.5944	2.0670	2.0219	2.2399	4.5288	1.0147
GC2	3.0732	2.0132	2.0687	2.2407	4.6353	0.8458
GC3	3.8112	2.0419	2.0389	2.2398	4.5667	0.9032
GC4	3.2853	1.5140	2.7538	2.4379	6.7134	1.2187

According to the literature^48,49^, the D and K of the semiconductor nanocrystals (NCs) depend on their crystal size. The thermodynamic rules of low dimensional materials suggest that a change of a size-dependent quantity is associated with the surface/volume ratio, or 1/x, where x is size of the nanocrystal. This suggests that x is the dominant factor in the heat transport of the semiconductor nanocrystals. The influence of crystal size on thermal transport is due to the surface scattering in the nanoparticle and phonon transport at the surfaces. The higher probability of diffusive scattering from the rougher surface may result in lower the thermal diffusivity (D). As seen from Table 3, the D for the GCs decrease (except in GC4) when compared with the G sample which means that in the latter the heat transportation is enhanced by the metallic ions concentration. In GCs, the thermal diffusivity behavior is dictated by the DMS nanostructures. The higher D of GC4 sample indicates excess of Ni content in the glass leading to a “metallization” of the system.^19^.

The relation between D and K may be given in terms of K = ${\rho }$cD, where ${\rho }c$ is the specific heat capacity. At room temperature, heat transport in solids is performed predominantly by phonons. Thus, the specific heat capacity is function of the phonon density of states. One of the ways to determine $({\rho }c)$ of the samples is via the use of the thermal relaxation technique^46,47^ as follows. An Ar$^+$ laser (60 mW and 488 nm) is used as heating source to the sample into $10^{-2}$ Torr atmosphere. After certain time, the laser is turned off. The cooling of the system is monitored. The result is presented in Fig. 5b. The fit of the experiment can be conducted in the heating or in the cooling process. We believe that the cooling process is more reliable due to the absence of external noise that may interfere into the measurement. As seen in Fig. 5b, the solid line indicates the theoretical fit to the equation (5) of the article of Anjos et al^46^, resulting in thermal relaxation time, ${\tau }$, given in seconds. Using the ${\tau }$ parameter, the $({\rho }c)$ is determined from, ${\tau }$ =${({\rho }cl_s)/(8{\sigma }T_0^3)}$, where ${l_s}$ is the thickness of the sample, ${\sigma }$ is the Stefan-Boltzmann constant and $T_{0}$ is the initial temperature. Table 3 shows the results of $({\rho }c)$ and K for all samples^19^.

The thermal conductivity (K) is comparable with some reported glasses. As reported by the Ghoneim and Halawa^50^, for instance, the thermal conductivity for the ${\text{Na}_2\text{O} + \text{B}_2\text{O}_3+\text{SiO}_2}$ glasses are higher and increase with ${\text{B}_2\text{O}_3}$ and ${\text{SiO}_2}$ content, indicating the formation of a network structure consisting of ${\text{BO}_4}$ and ${\text{SiO}_4}$ tetrahedral units. This results in a longer phonon mean free path and consequently in a higher thermal conductivity. Similar phenomenon is thought to occur in our samples, i.e., an increase of the ordering of the glass network structure by “metallization” or through network formation of DMS nanostructures resulting in longer phonon mean-free-path. This behavior is traduced via the formula, K = ${{\rho }c{\nu }_sl_{p}/3}$, where K is the thermal conductivity at room temperature and ${\nu }_s$ is the average sound velocity and $l_{p}$ is the phonon mean free path^51^. The behavior of D and K of our samples are presented in Fig. 5c.

The temperature variation of the optical path, dS/dT, is another important parameter for operating devices in practical temperatures which should require low dS/dT. This parameter is generally difficult to be controlled by the glass composition since ${dS/dT = n{\alpha } + dn/dT}$. From TL experiments, one can estimate dS/dT with the knowledge of the TL-induced phase shift through the following expression^45^:

3 $$\begin{aligned} \theta = -\frac{P_{abs}}{K\lambda _p} {\varphi }\frac{dS}{dT}, \end{aligned}$$

in which, ${\lambda _p}$ is the probe beam wavelength and $P_{abs}$ is the absorbed pump power. The ${P_{abs} = P_{ex}A_{e}l_{s}}$, where $P_{ex}$ is the excitation beam power, $l_{s}$ is the sample thickness, and $A_{e}$ is the optical absorption coefficient of the sample. K is the thermal conductivity and ${\varphi }$ is the fraction of absorbed energy converted into heat (${\varphi }$ = 1 in the present work). The low dS/dT values for G and GCs (see Table 3) are attributed to the occurrence of laser beam deviation inside the samples. It is worth to mention that our samples are not completely homogeneous thus the reported values were average of five measurements. The GC2 shows the lower dS/dT among the studied glasses.

### Third-order nonlinear properties

**Figure 6:**
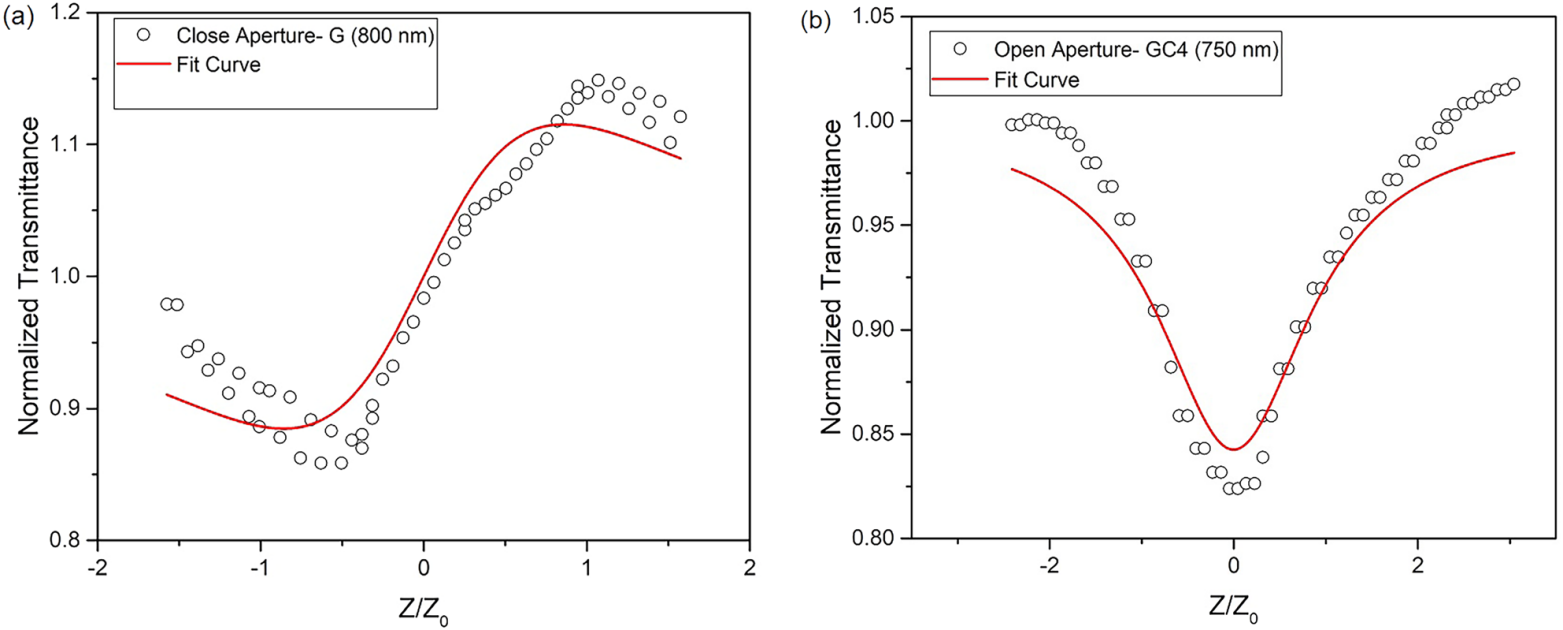
Z-scan results in the close-aperture mode for the sample 5% Ni at 800 nm (**a**) and the open-aperture mode for sample 10% Ni at 750 nm (**b**).

Generally, the nonlinear optical properties of the material varies in function of the excitation wavelength, peak intensity, pulse width and excited state lifetime^52^. In this work, the third-order nonlinear optical (NLO) parameters such as the nonlinear refraction $({n_2})$ (close-aperture, CA) and nonlinear absorption coefficient $({\beta })$ (open-aperture, OA) were measured using femtosecond Z-scan experiment with excitation wavelengths, 750, 800 and 850 nm^19^. Figure 6a shows CA traces that allowed us to find the sign and magnitude of ${n_2}$ in 5% Ni doped glass.

It is observed that the peak followed a valley configuration indicating the self-focusing behavior under high laser irradiance, namely, a positive sign of ${n_2}$ which can be calculated from the transmittance gap between the peak and valley by fitting the CA Z-scan through the following equation^53^

4 $$\begin{aligned} T(Z) = 1 - \frac{4x{\Delta }{\phi }}{\sqrt{2(x^2+1)(x^2+9)}} \end{aligned}$$

where x = ${Z/Z_0}$, Z is the longitudinal distance from the focal point, ${Z_0}$ is the Rayleigh range of the beam, and ${\Delta \phi }$ is the nonlinear phase change. The obtained value of ${\Delta }{\phi }$ by the experimental fit is used to calculate ${n_2}$ by,

5 $$\begin{aligned} n_2 = \frac{{\Delta }{\phi }{\lambda }{\alpha }}{2{\pi }I_{0}L_{eff}}, \end{aligned}$$

where ${\alpha }$ is the linear absorption coefficient at 800 nm, ${L_{eff}}$, is the effective thickness of the sample, ${I_0}$ is the peak intensity at the focus and ${\lambda }$ is the wavelength of the pump laser. The calculated ${n_2}$ is 3.54 ${\times }$ 10$^{-14}$ cm$^2$/W, which is nearly two orders lower than those of the Ni doped chalcogenide glasses^54^ and CsPbBr$_{3}$ with NCs^55^. The measurement of ${n_2}$ using CA Z-scan technique did not give detectable changes of post (pre)-focal transmittance intensity within the measured sensitivity and intensity (wavelength) range for glass samples containing Ni-doped NCs.

Two GC samples show nonlinear absorption using OA Z-scan, i.e., GC2 (1.0% Ni) at wavelength 800 & 850 nm, and GC4 (10.0% Ni) at wavelength 750 & 800 nm. Figure 6b shows OA Z-scan traces as an example for the 1.0% (${\lambda }$ = 850 nm) and 10% (${\lambda }$ = 750 nm) Ni doped GCs. An inverted-bell shaped transmittance with a minimum at focus, Z = 0 supports absorption saturation. The transmittance data was theoretically fitted to the nonlinear transmittance equation for the two-photon absorption (TPA) process^53^,

6 $$\begin{aligned} T(Z) = 1 - \frac{{\beta }I_0L_{eff}x}{2\sqrt{2}{(x^2+1)}} \end{aligned}$$

The calculated TPA coefficent $({\beta })$ for the GC2 is 2.53 ${\times }$ 10$^{-10}$ cm/W (${\lambda }$ = 800 nm) and 2.76 ${\times }$ 10$^{-10}$ cm/W (${\lambda }$ = 850 nm); and for GC4 is 7.98 ${\times }$ 10$^{-10}$ cm/W (${\lambda }$ = 750 nm) and 7.35 ${\times }$ 10$^{-10}$ cm/W (${\lambda }$ = 800 nm), respectively. We noted that the ${\beta }$ does not change significantly with wavelength, but increase with Ni concentration due to the bound electronic effects and TPA. The laser energy $({\hbar }{\omega })$ used in this work meet the TPA condition, ${E_g}$ < 2${\hbar }{\omega }$ < $2{E_g}$, where ${E_g}$ is the energy bandgap. Therefore, the small decrease of ${E_g}$ (3.77 to 3.72 eV) leads to significant increase of ${\beta }$ for samples, GC2 to GC4. This fact is supported by Dinu’s theoretical model for semiconductor glasses (i.e, ${\beta }$ ${\propto }$ ${1/({E_g})^3}$)^56^. However, one can not neglect the quantum confinement effects because the existence of interband transitions (2.26 eV) (see Fig. 2a) attributed to the ZnTe NCs in GC2 and GC4 samples. The increase of the nonlinear absorption coefficient is a result of the increase of the oscillator strength caused by the confinement-induced localization of the excitations. In fact, the exciton Bohr radius $({a_0})$ decreases with the size of ZnTe NCs with respect to the bulk. Thus the third-order nonlinear optical property increases proportionally to $(1/{a_0})$^57^. For comparison, the obtained ${\beta }$ values in studied samples are higher than the values of zinc phospho-tellurite glass containing ZnTe quantum dots (0.06–0.23 ${\times }$ 10$^{-10}$ cm/W, ${\lambda }$ = 800 nm)^58^, silicate glass containing Ni NPs (0.239 ${\times }$ 10$^{-10}$ cm/W, ${\lambda }$ = 800 nm)^59^ and Ni doped CsPbBr$_{3}$ NCs (0.38–1.0 ${\times }$ 10$^{-10}$ cm/W, ${\lambda }$ = 800 nm)^55^, respectively.

## Conclusions

In summary, the structural and thermo-optical properties of phosphate glass (G) doped only with Ni ions and phosphate glass containing semiconductor nanocrystals (GCs) were studied. From XRD measurements, the G sample exhibited glassy characteristic while the GCs samples exhibit not only the amorphous halo, but also sharp diffraction peaks that were attributed to the presence of ZnTe quantum dots (QDs) or nanocrystals (NCs). The distribution of phosphate groups, ${PO_2^-}$, ${PO_3^{2-}}$, ${PO_4^{3-}}$ and ${P_2O_7^{4-}}$ were discriminated through infrared absorption and Raman spectroscopy. The longitudinal optical (LO) and transverse optical (TO) phonon modes of ZnTe in GCs were observed through structural analysis. The average diameter of 4.2 ${\pm }$ 0.3 nm and 13.4 ${\pm }$ 0.2 nm for QD and bulk NCs were observed for the thermally annealed (for 10 h at 500 $^o$C) GC2 through TEM analysis.

The spin allowed and bulk ZnTe crystal electronic transitions were observed around 1366, 822, 434 nm and 533 nm. The band gap energy ${(E_{opt})}$ for direct and indirect transition were estimated from optical absorption edges. The observed decrease of ${E_{opt}}$ for the G sample compared to GCs indicates the increase of non-bridging oxygen’s (NBOs). Under 240 nm excitation, the emission and decay lifetime measurements were reported. The trend of emission intensity, FWHM and decay lifetimes were similar in GCs, which is a strong evidence of the change of the environment surrounding of Ni$^{2+}$ ions due to the presence of the ZnTe NCs. The ${\sigma _{emi}}$ increases with the increase of Ni$^{2+}$ content in GCs and it is significantly improved compared with the G sample. The highest value of FOM is observed in GC4 ${(11.06 \times 10^{-14} \; \text{cm}^2\;\text{s})}$ among the GCs.

Time-resolved thermal lens and thermal relaxation methods have been applied to determine the thermal diffusivity (D), variation of optical path length with temperature (dS/dT) and thermal conductivity (K) of the glass samples. D for the GCs decreased (except in 10% Ni doped GC) when compared with the G sample which means that the host glass dominates the heat transport due to the low concentration of DMS semiconductor NCs. In addition, the D and K does not change significantly with increase of Ni$^{2+}$ (0.5–5.0 wt%) content in GCs. GC4 shows higher nonlinear absorption coefficient ${(7.98 \times 10^{-10} \; \text{cm}^2/\text{W})}$ among the studied samples. Finally, due to the fact that GC4 sample has jointly low dS/dT, high emission cross-section and has the highest FOM between the samples definitely suggests this sample as suitable for visible-red light conversion in LED technology.

## Materials and methods

Phosphate glass composition, 65P$_2$O$_5$ + 14ZnO + 1Al$_2$O$_3$ + 10BaO + 10PbO (mol %) doped with 5 wt% of Ni$^{2+}$ ions and doped with Te (1 wt%) and x-Ni content (wt%) varying at an expense of Zn (x = 0.5, 1.0, 5.0 and 10) for the formation of the semiconductor nanocrystals, were prepared by fusion method. The prepared samples are named as G for glass doped only with 5 wt% Ni$^{2+}$ ions; GC1, GC2, GC3 and GC4 for the glass containing nanocrystals and doped with increasing Ni-content (0.5, 1.0, 5.0 and 10.0 wt%), respectively. The preparation method is similar to the one described in literature^17,18,20,23^. The batch compositions were melted in an alumina crucibles at 1300 $^{\circ }$C at 30 min under carbon-rich atmosphere, which is of fundamental importance since it reduces oxidation of the glass matrix precursors. This reduction further increases the concentration of available Zn$^{2+}$, Te$^{2-}$ and Ni$^{2+}$, which is necessary for the formation and growth of Zn$_{1-x}$Ni$_x$Te NCs. Next, these melted mixtures were quickly quenched, forming a glass system doped with the precursor ions. Posteriorly, the glass samples were thermally annealed at 500 $^{\circ }$C for 10 h to enhance the diffusion of Zn$^{2+}$, Te$^{2-}$ and Ni$^{2+}$ and induce growth the of NCs.

To obtain the DTA thermograms, a Shimadzu DTA-50 analyzer was used with a heating rate of 20 $^{\circ }$C/min. Thus, it was possible to determine the glass transition temperature (T$_g$) of the glass samples with an uncertainty of ${\pm }$5 $^{\circ }$C. XRD patterns were recorded using a XRD-6000 Shimadzu diffractometer equipped with monochromatic CuK${\alpha }$1 radiation (k = 1.54056 Å) and set to a resolution of 0.02. Scanning transmission electron micrographs were taken using a JEOL, JEM-2100, 200 kV microscope to investigate the formation and size of the NCs. For these measurements, 50 mg of powder sample were diluted in isopropyl alcohol and subjected to ultra-sound for 15 min. After one minute, an aliquot of the supernatant was dripped on a thin film of copper coated with carbon. After drying, the film containing the sample was placed in the measurement equipment. Infrared reflectance spectral measurements in the wavenumber range 1500–400 cm$^{-1}$ were recorded using FT-IR-ATR, VERTEX 70 (BRUKER) spectrophotometer. Raman spectra were recorded with micro-Raman (HORIBA JOBIN IVON T64000) operating at 488 nm in the wavenumber range 200-1500 cm$^{-1}$. Optical absorption spectra show optical transition bands in the wavelength range 190–900 nm and 1080–2450 nm. These were measured on UV 2500 (SHIMADZU) and FT-NIR (MPA—Multi Purpose Analyzer, BRUKER) spectrophotometers, respectively. Excitation, emission and decay measurements of the samples has been recorded using FS920 spectrometer (Edinburgh Instruments) equipped with 450W Hydrogen Arc Lamp and a single photon counting photomultiplier (Hamamatsu R2658P).

The TL technique used in this work is the dual beam mismatched mode configuration reported in the literature^46^. He-Ne laser at 632.8 nm was used as probe beam and an Ar$^{+}$ laser at 488 nm was used as pump beam. The pump and probe beams are detected by two silicon photodiodes connected to a Tektronix TDS2020 digital oscilloscope. Heat capacity measurements were performed according to thermal relaxation method (TRM)^46^. A thin carbon film is deposited on the samples and is thermally isolated in a vacuum sealed Dewar flask. One of its faces is heated with an Ar$^{+}$ laser at 488 nm (60 mW) and the temperature of the sample was recorded as a function of time via an infrared sensor placed near (1 mm) the rear surface of the sample. Afterwards, the laser was blocked and a cooling curve was measured.

The third order nonlinear optical measurements were carried through an ultrafast Z-scan setup. A mode-locked Ti: sapphire laser (Mai Tai-Spectra Physics) was used with 750 to 850 nm wavelength range with a repetition rate of 80 MHz and pulses widths of 100 fs. Then the laser beam is focused by lens with 15 cm focal length and the sample moves along the propagation direction (z) of the laser beam by a translation stage (Newport) while a Si-photodetector records the transmitted beam far from the focal point of the lens. An aperture is placed just before the detector. The open/close aperture Z-scan was used to measure the magnitude and sign of the nonlinear absorption coefficient ($\beta$), and nonlinear refractive index (n$_2$). All these measurements were performed at room temperature. The complete experimental procedure and setup is described previously^60^.

## Supplementary Information

Supplementary Information.

## Data Availability

The data sets generated during and/or analysed during the current study are available from the corresponding author on reasonable request.
