# Supplementary material for: Zn1-xNixTe semiconductor nanocrystals in transparent glass for optoelectronic device applications
Source: Sci Rep. 2023 May 10;13:7627. doi: 10.1038/s41598-023-34591-0 (PMC10172339; doi:10.1038/s41598-023-34591-0)
Supplement: Supplementary file 1 — Supplementary Information. [file 41598_2023_34591_MOESM1_ESM.docx]

SUPPORTING INFORMATION

# Zn_1-x_Ni_x_Te semiconductor nanocrystals in transparent glass for optoelectronic device applications

**Radha Mada^1*^, Hamid Darabian^1^, Seshadri Meruva^1^, Maria Jose V. Bell^1^, Virgilio C. Anjos^1*^, Alessandra S. Silva^2^, and Noelio O. Dantas^2^**

**^1^**Grupo de Engenharia e Espectroscopia de Materiais, Departamento de Fisica – ICE, Universidade Federal de Juiz de Fora, Juiz de Fora – MG 36036-900, Brazil.

^2^Laboratorio de Novos Materiais Nanoestruturados e Funcionais, Instituto de F´ısica, Universidade Federal de Alagoas, Macei´o - AL, 57072-900, Brazil.

*Corresponding authors E-mail: [radha.physics@ice.ufjf.br](mailto:radha.physics@ice.ufjf.br) (M. Radha); [virgilio.anjos@ufjf.edu.br](mailto:virgilio.anjos@ufjf.edu.br) (V. Anjos); Tel.: [+55-32-2102-3307 Ext: 237](tel:+55%2032%202102-3307)  (office) or 234 (lab)

### **Results and discussion**

### **Optical absorption and emission spectroscopy**


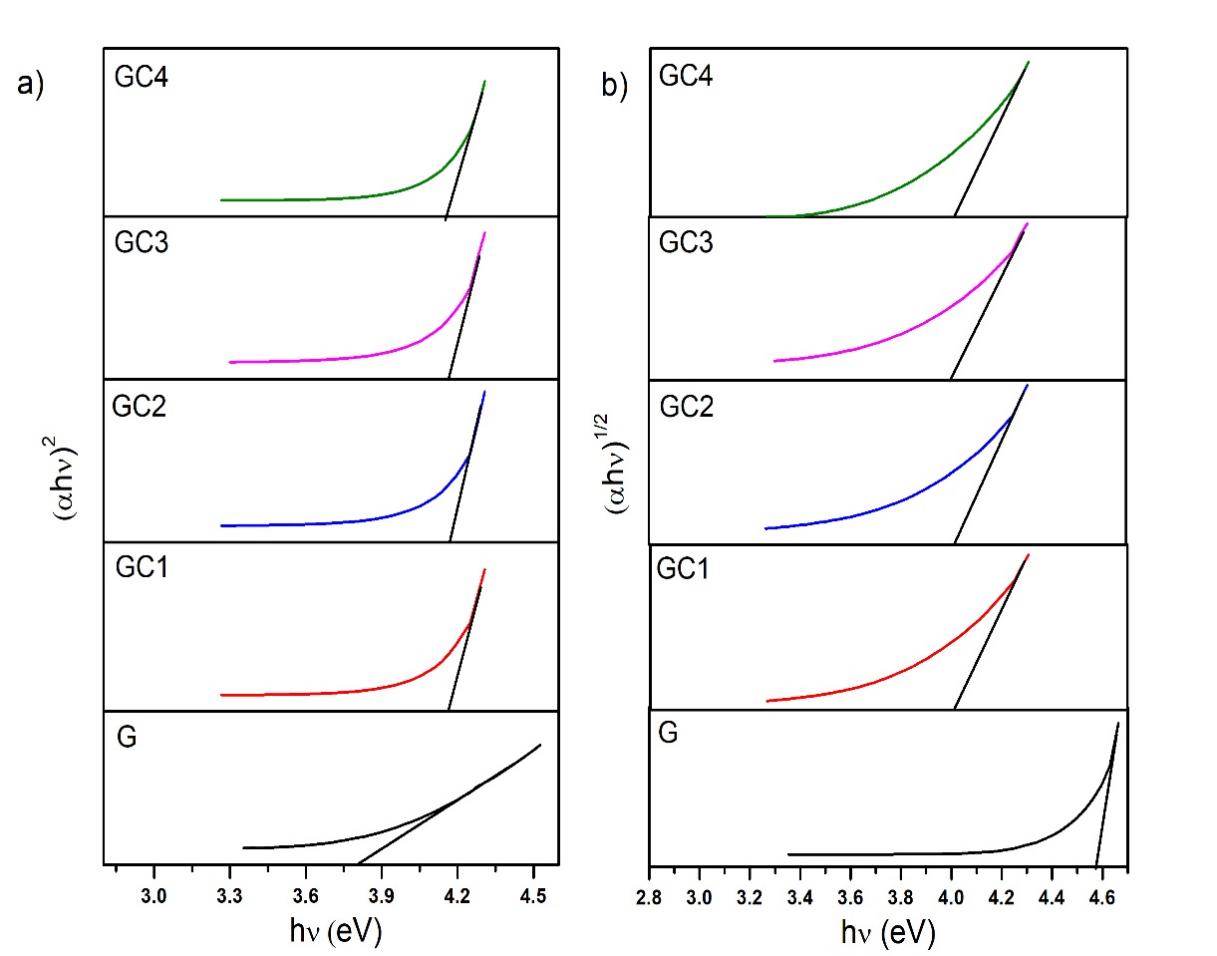


Figure S1. Plots of ${(\alpha h)}^{2}$ as a function of $h$ (a), and ${(\alpha h)}^{1/2}$ as a function of $h$ (b) for the Ni^2+^ contain samples.

According to Davis and Mott [35] theory, the optical energy band gap of an amorphous material is estimated through its ultraviolet absorption edge with the following expression,

$\alpha\left( \omega\right)=A\frac{{(\hbar\omega-E_{\mathrm{opt}})}^{n}}{\hbar\omega} .$

In the above equation, the exponent n = 1/2 represents an allowed direct transitions, while n = 2 represents an indirect one, A is a constant, E_opt_ is the optical band gap and ℏω the photon energy of the incident radiation. The α(ω) is the absorption coefficient obtained from the absorbance spectra. Using above expression, by plotting ${(\alpha h)}^{2}$ and ${(\alpha h)}^{1/2}$ as a function of $h$ (see fig. S1(a) and S1(b)) near the absorption edges).
